# Mechano-biological adaptation of the pulmonary artery exposed to systemic conditions

**DOI:** 10.1038/s41598-020-59554-7

**Published:** 2020-02-17

**Authors:** Emma Vanderveken, Julie Vastmans, Piet Claus, Eric Verbeken, Heleen Fehervary, Lucas Van Hoof, Katrien Vandendriessche, Peter Verbrugghe, Nele Famaey, Filip Rega

**Affiliations:** 1 Department of Cardiovascular Sciences, KU Leuven, Leuven, Belgium; 2 Department of Mechanical Engineering, KU Leuven, Leuven, Belgium; 3 Department of Imaging and Pathology, KU Leuven, Leuven, Belgium; 4 Department of Cardiac Surgery, University Hospitals Leuven, Leuven, Belgium

**Keywords:** Interventional cardiology, Biomedical engineering

## Abstract

Cardiac surgeries may expose pulmonary arterial tissue to systemic conditions, potentially resulting in failure of that tissue. Our goal was to quantitatively assess pulmonary artery adaptation due to changes in mechanical environment. In 17 sheep, we placed a pulmonary autograft in aortic position, with or without macroporous mesh reinforcement. It was exposed to systemic conditions for 6 months. All sheep underwent 3 ECG-gated MRI’s. Explanted tissue was subjected to mechanical and histological analysis. Results showed progressive dilatation of the unreinforced autograft, while reinforced autografts stabilized after two months. Some unreinforced pulmonary autograft samples displayed more aorta-like mechanical behavior with increased collagen deposition. The mechanical behavior of reinforced autografts was dominated by the mesh. The decrease in media thickness and loss of vascular smooth muscle cells was more pronounced in reinforced than in unreinforced autografts. In conclusion, altering the mechanical environment of a pulmonary artery causes changes in its mechano-biological properties.

## Introduction

Various surgical interventions result in the exposure of the pulmonary artery to aortic conditions. For example, during the Ross procedure, the diseased aortic root is replaced by the pulmonary root^1–3^. Children born with hypoplastic left heart syndrome undergo the Norwood procedure in order to create a neoartic root from the pulmonary artery^4^. As a final example, the pulmonary artery and aorta are surgically switched in patients with transposition of the great arteries, placing the pulmonary root in the systemic circulation^5–7^. Often, failure of these procedures is related to inability of the pulmonary artery to adapt to the systemic conditions^4,8^. Various reinforcement strategies with synthetic materials such as Dacron, and more recently, with a macroporous mesh, are proposed and implemented in clinical practice.

Despite their similar embryological origin^9^, the aorta and the pulmonary artery show critical differences. There are three key hemodynamic differences between the aorta and the pulmonary artery: blood pressure (120/80 mmHg vs. 8–20 mmHg), peak blood flow velocity (92 ± 11 cm/sec vs. 63 ± 9 cm/sec) and blood flow acceleration rate (940 ± 161 cm/sec^2^ vs. 396 ± 70 cm/sec^2^)^7^.

On a mechanical level, several studies show that pulmonary artery is more compliant than aorta^10,11^. The opposite was found when testing pulmonary artery and aorta biaxially^12^. Differences in tissue origin, age of the samples and testing methods explain these contradictory results.

There is more consensus on a histological level. Elastin, collagen and smooth muscle cells (SMC) are the main building blocks of both arteries, albeit with differences in quantity and organization. The aortic wall thickness is higher as well as more uniform. Furthermore, the aorta contains a greater amount of elastic lamellae, packed into a denser weave, and collagen is more regularly distributed^12,13^.

Many efforts are undertaken to model the adaptation processes that take place after exposing pulmonary artery to a new mechanical environment^14^. Studies examining the remodeling of failed pulmonary autografts, as well as studies investigating arterial remodeling following hypertension, mention an increased extracellular matrix deposition and, consequently, a change in mechanical properties^2,8,15–21^. To our knowledge, quantitative experimental data on both the mechanical and histological adaptation of arterial tissue to external mechanical stimuli in a large animal model are currently non-existent.

**Figure 1:**
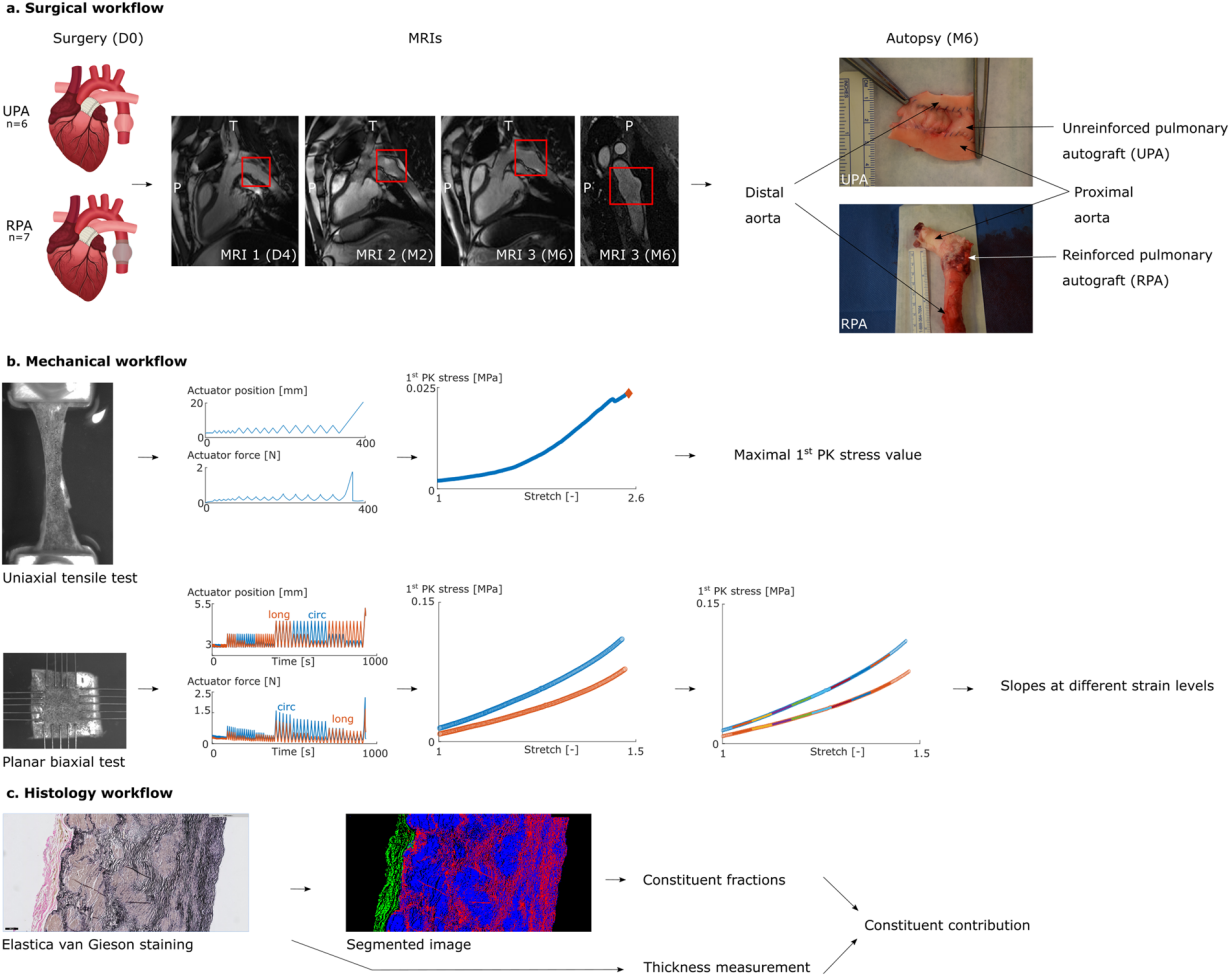
Workflow. The pulmonary artery was reimplanted as an interposition graft in aortic position and reinforced in half of the cases (D0). 6 UPA sheep and 7 RPA sheep were included. Follow-up consisted of three ECG-gated MRIs (D4, M2 and M6). After 6 months of implantation, samples were analyzed both mechanically and histologically. Planar biaxial and uniaxial tensile testing resulted in valuable information on the tissue stiffness and strength. Histological analysis consisted of quantifying the wall thickness and the constituent contribution. Copyright holder: Brecht vanderveken.

The goal of this study was to assess the remodeling of the pulmonary artery as a response to a changed mechanical environment, *in casu* as an interposition autograft in aortic position. We assessed two conditions: one in which the autograft was reinforced with a macroporous mesh (9 sheep, reinforced pulmonary autograft (RPA) group) and one without reinforcement (8 sheep, unreinforced pulmonary autograft (UPA) group). Follow-up consisted of three ECG-gated MRI’s over 6 months in which the diameter of the aorta and autograft was tracked. Afterwards, the acquired tissues were subjected to mechanical analysis (*i.e*. planar biaxial testing for stiffness and nonlinear stiffening and uniaxial testing for tissue strength), and histological analysis (quantification of collagen, elastin and SMC) (Fig. 1).

## Results

Two sheep died in the unreinforced group, one after dehiscence of the anastomosis between the aorta and pulmonary autograft at day 11, the second due to right ventricular failure during the first MRI. Likewise, two sheep died in the reinforced group, both due to surgical complications. These four were excluded from further analysis.

The mean animal weight increased from 44 ± 2 kg to 59 ± 5 kg in the UPA group and from 44 ± 4 kg to 58 ± 6 kg in the RPA group during the six months after surgery.

In the UPA group, the axial prestretch imposed on the pulmonary autograft, *i.e*. the length after implantation divided by the length before explantation, was 1.1 ± 0.1 (measured only on 4 sheep, outlier of 2.9 excluded). The pulmonary autografts in the RPA group had a mean axial prestretch of 1.3 ± 0.2 before placing the macroporous mesh (measured on 6 sheep).

### Pulmonary autograft dimensions

**Figure 2:**
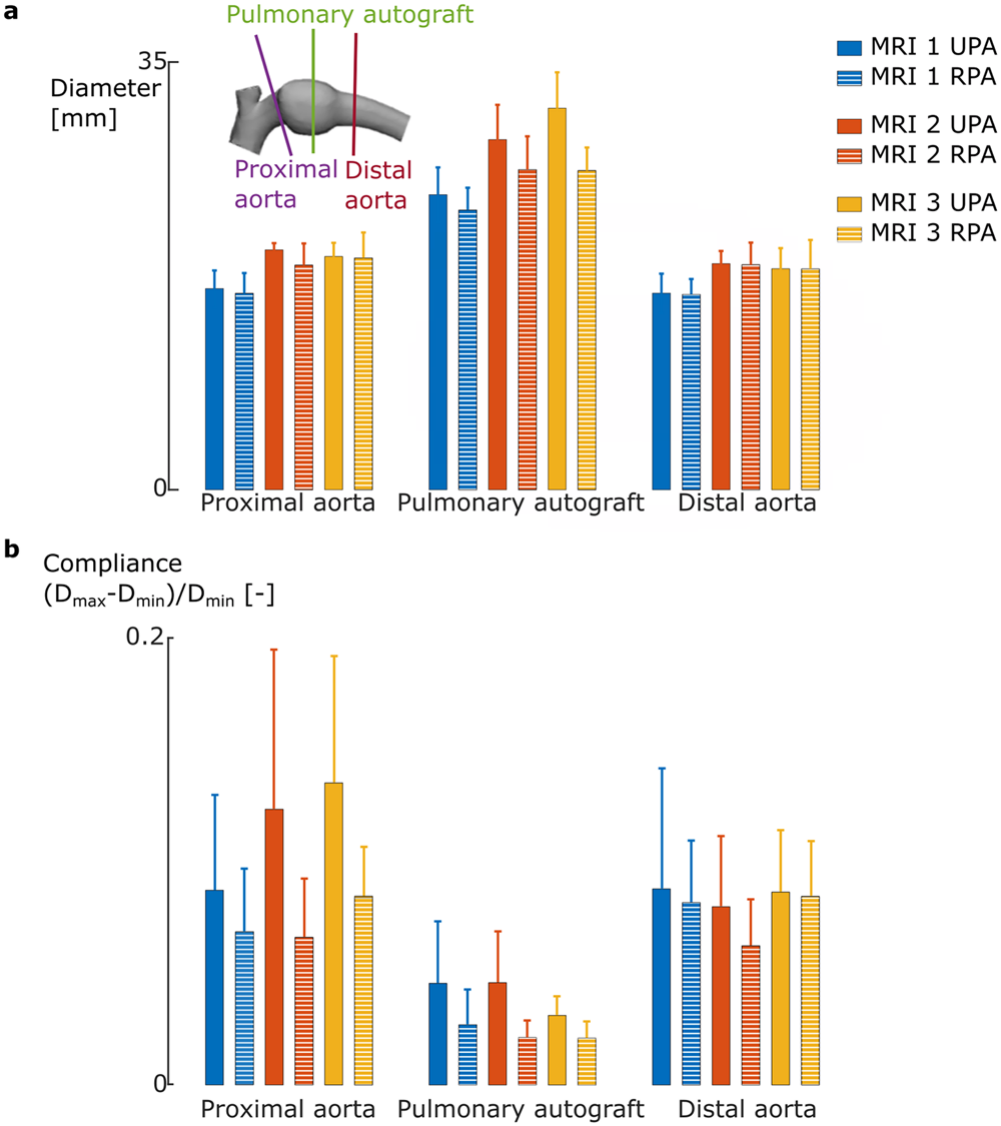
Reinforcement halts unlimited dilatation of the pulmonary autograft but reduces *in vivo* compliance. **(a)** Graph representing the mean diameter data for the proximal aorta, pulmonary autograft and distal aorta over time for both the UPA and RPA group. The mean autograft diameter increases in the UPA group for the three timepoints, whereas the mean diameter of the reinforced autograft stabilizes after two months. **(b)** The difference between the maximal and minimal diameter divided by the minimal diameter, measured on the different MRIs for the different tissues for the UPA and RPA sheep. In the UPA group, the compliance of the autograft is lower than the compliance of the aortic tissues and decreasing over time. When adding a macroporous mesh, the low compliance compared to aortic tissue is further enhanced.

The growth of the proximal and distal aorta is similar between both groups. The mean UPA diameter keeps expanding (MRI 1: 24.2 ± 2.0 mm vs. MRI 3: 31.2 ± 2.6 mm (p = $2.9{e}^{-10}$)). In the reinforced sheep, the initial dilatation seems to stagnate two months after surgery (Fig. 2a).

The difference between the maximal (*i.e*. systolic) and minimal (*i.e*. diastolic) diameter divided by the minimal diameter is taken as a measure for compliance (Fig. 2b). The difference in compliance between proximal and distal aorta is more pronounced for the UPA group. The compliance of the UPA is lower than that of the native aorta, both proximal (p = 0.07) and distal (p = 0.7). Additionally, the compliance of the UPA decreases over time (MRI 1: 0.05 ± 0.02 vs. MRI 3: 0.03 ± 0.01, p = 0.15). Adding the reinforcement decreases the initial compliance of the RPA (MRI 1: 0.03 ± 0.01, p = 0.16) compared to the UPA. However, the compliance of the RPA remains stable throughout the six months (MRI 3: 0.02 ± 0.01).

### Mechanical analysis

**Figure 3:**
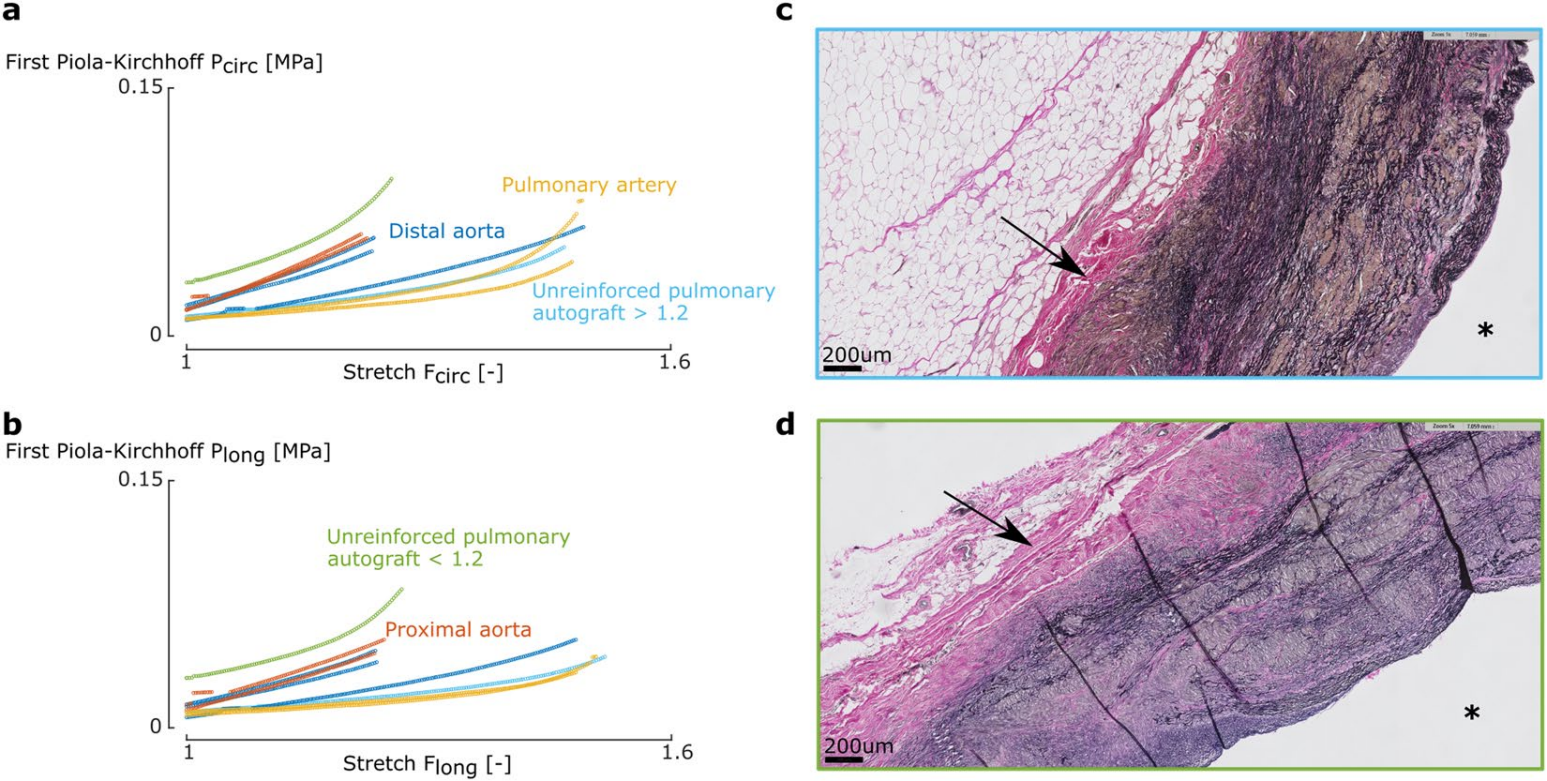
Different behavior between and within pulmonary autografts is visible both mechanically and structurally. **(a,b)** Representative stress-stretch curves of biaxial samples of one UPA sheep in the circumferential **(a)** and longitudinal **(b)** direction for the 1:1 ratio. The graphs show a distinctly different behavior for pulmonary artery compared to distal and proximal aorta, with an initially lower slope of the stress-stretch curves of the former. In the higher stretch region, the stiffening effect, *i.e*. the sudden increase in the curve, is more clear for native pulmonary artery. Two types of pulmonary autograft samples are present: on the one hand samples that reach a high stretch and do not show remodelled behavior compared to native pulmonary artery (light blue line), and on the other hand samples that do not reach a stretch of 1.2 and display remodelled behavior acting more aorta-like (green line), reflected in a higher collagen deposition as seen in **(d)**. **(c,d)** Transverse microscopic sections of UPA representing the difference in collagen deposition. Elastica Van Gieson stain, original magnification x5. The luminal side is marked with an asterisk. The difference in mechanical behavior of the UPA samples is visible in the wall composition, *i.e*. an increase in collagen deposition (**d**) or no collagen changes (**c**) (black arrows).

**Figure 4:**
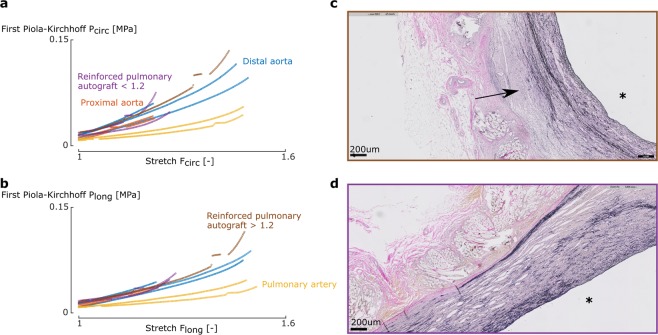
Degree of mesh incorporation affects the mechanical behavior of the pulmonary autograft - mesh complex. **(a,b)** Representative stress-stretch curves of biaxial samples of one UPA sheep in the circumferential **(a)** and longitudinal **(b)** direction for the 1:1 ratio. When incorporated well **(d)**, the mechanical behavior of the RPA is mainly dominated by the reinforcement (purple line) and is not able to reach a stretch higher than 1.2. A proliferating, but inactive SMC population in some cases (black arrow) and edema in other cases is present between the mesh and the vessel wall due to an inadequate mesh fit **(c)**, potentially leading to detachment of the mesh during testing and allowing the sample to be stretched further. **(c,d)** Transverse microscopic sections of RPA. Elastica Van Gieson stain, original magnification x5. The luminal side is marked with an asterisk.

**Figure 5:**
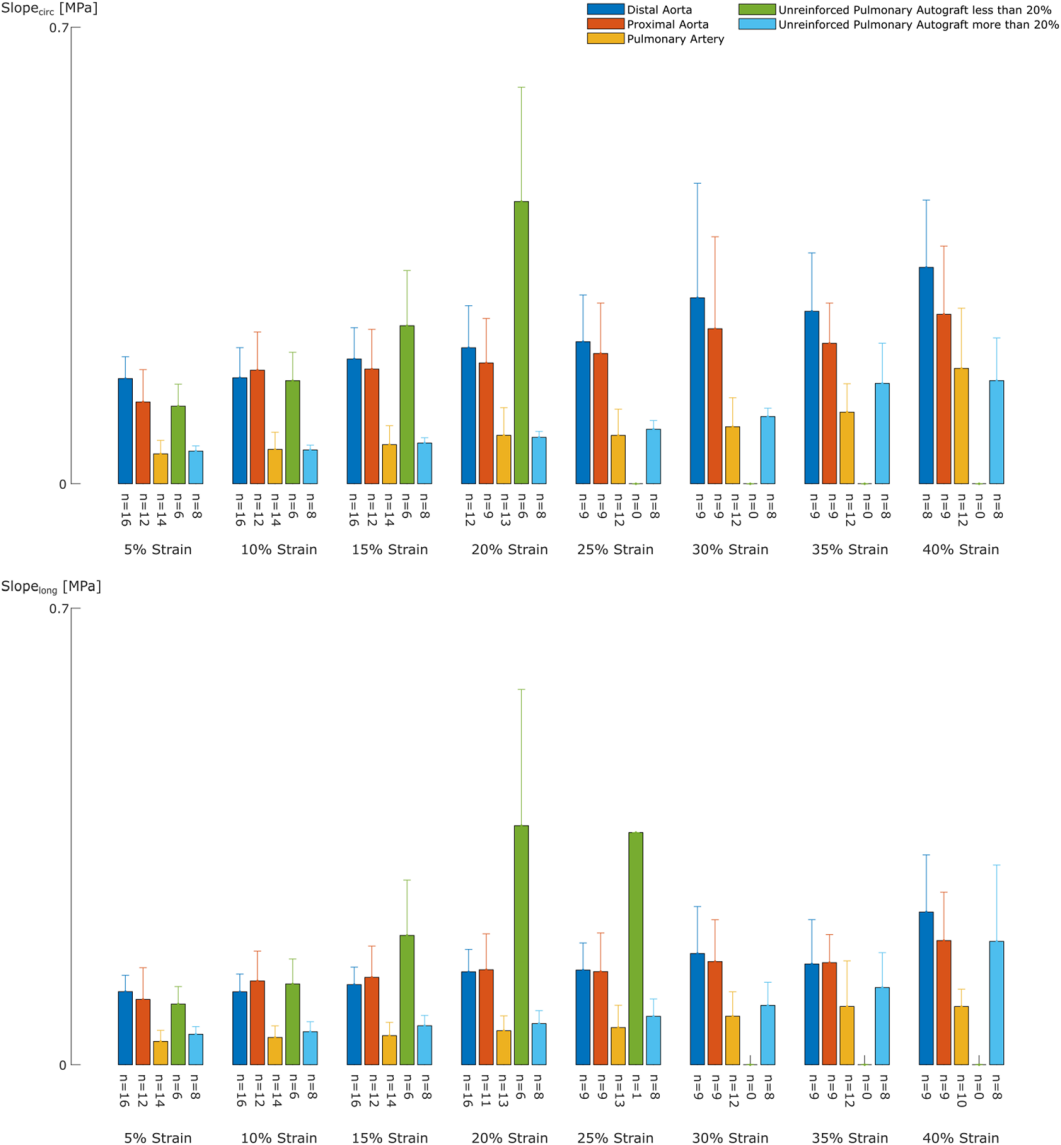
The UPA displays two types of mechanical behavior. The graphs represent the average slopes of the stress-stretch curves of the UPA sheep at the different stretch levels for the circumferential (top) and longitudinal (bottom) direction. We can distinguish two groups within the UPA samples: the samples that stiffen at low strain (green), showing more aorta-like behavior and the samples that stiffen at high strains (light blue), showing more pulmonary-like behavior.

**Figure 6:**
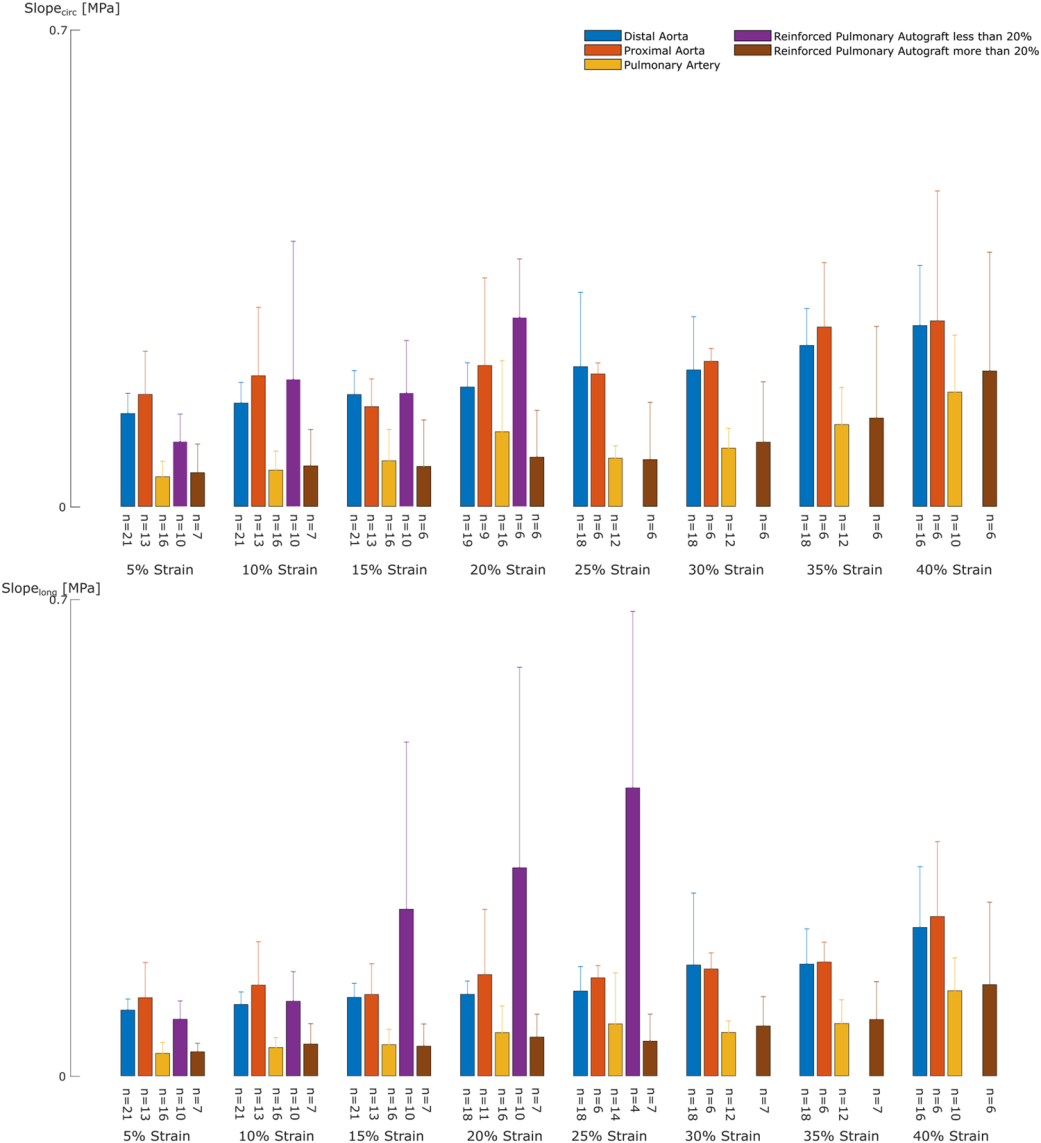
The RPA displays two types of mechanical behavior. The graphs represent the average slopes of the stress-stretch curves of the RPA sheep at the different stretch levels for the circumferential (top) and longitudinal (bottom) direction. We can distinguish two groups within the RPA samples: the samples that stiffen at low strains (purple) and the samples that stiffen at high strains (brown). We postulate that this difference arises from good incorporation of the mesh in the former group in which the mechanical behavior is dominated by the reinforcement and bad incorporation in the latter group, showing more native pulmonary-like behavior.

A representative set of samples for a sheep of the UPA and RPA group is shown in Figs. 3 and 4. The slopes of all stress-stretch curves at the different stretch levels are given in Fig. 5 for the UPA group and in Fig. 6 for the RPA group. In both groups, the distal and proximal aorta are stiffer than the pulmonary artery at the same stretch level in both directions. However, the increase in slope at higher stretch levels, a proxy for nonlinear stiffening behaviour, is more distinct for the pulmonary artery compared to the aortic samples.

For the UPA group, two categories of pulmonary autograft samples could be made, based on the stretch level at which the samples failed in the biaxial test. Both sample types could be found within the same sheep. The samples that failed at a stretch lower than 1.2 are stiffer at low stretch levels than their counterparts which failed at higher strains. Also, the nonlinearity is much higher in the former. For the RPA group, again two groups were distinguishable: samples that stiffen at lower strains and do not reach a stretch of 1.2 and samples that stretch further and stiffen at high strains. In the former, the reinforced autografts are stiffer than the pulmonary artery, but more compliant than the aortic samples. No difference between the latter group and native pulmonary artery samples was noticeable.

The maximally measured ${1}^{st}$ Piola-Kirchhoff stress during the uniaxial tensile test, both in circumferential and longitudinal direction, serves as a proxy for the tensile strength. For the aortic and pulmonary artery samples, the circumferential tensile strength is significantly higher than the strength in the longitudinal direction (distal aorta: 20.5 ± 7.0 kPa vs. 65.5 ± 20.7 kPa (p = $7.7{e}^{-10}$), proximal aorta: 22.4 ± 7.6 kPa vs. 64.0 kPa ± 24.4 kPa (p = $0.00037$) and pulmonary artery: 24.0 ± 21.8 kPa vs. 30.6 kPa ± 11.9 kPa (p = 0.4)). When looking at the autograft samples, it is evident that adding the reinforcement increases the longitudinal tensile strength (16.9 ± 3.7 kPa vs. 37.0 ± 7.4 kPa (p = 0.009)).

### Histological analysis

**Figure 7:**
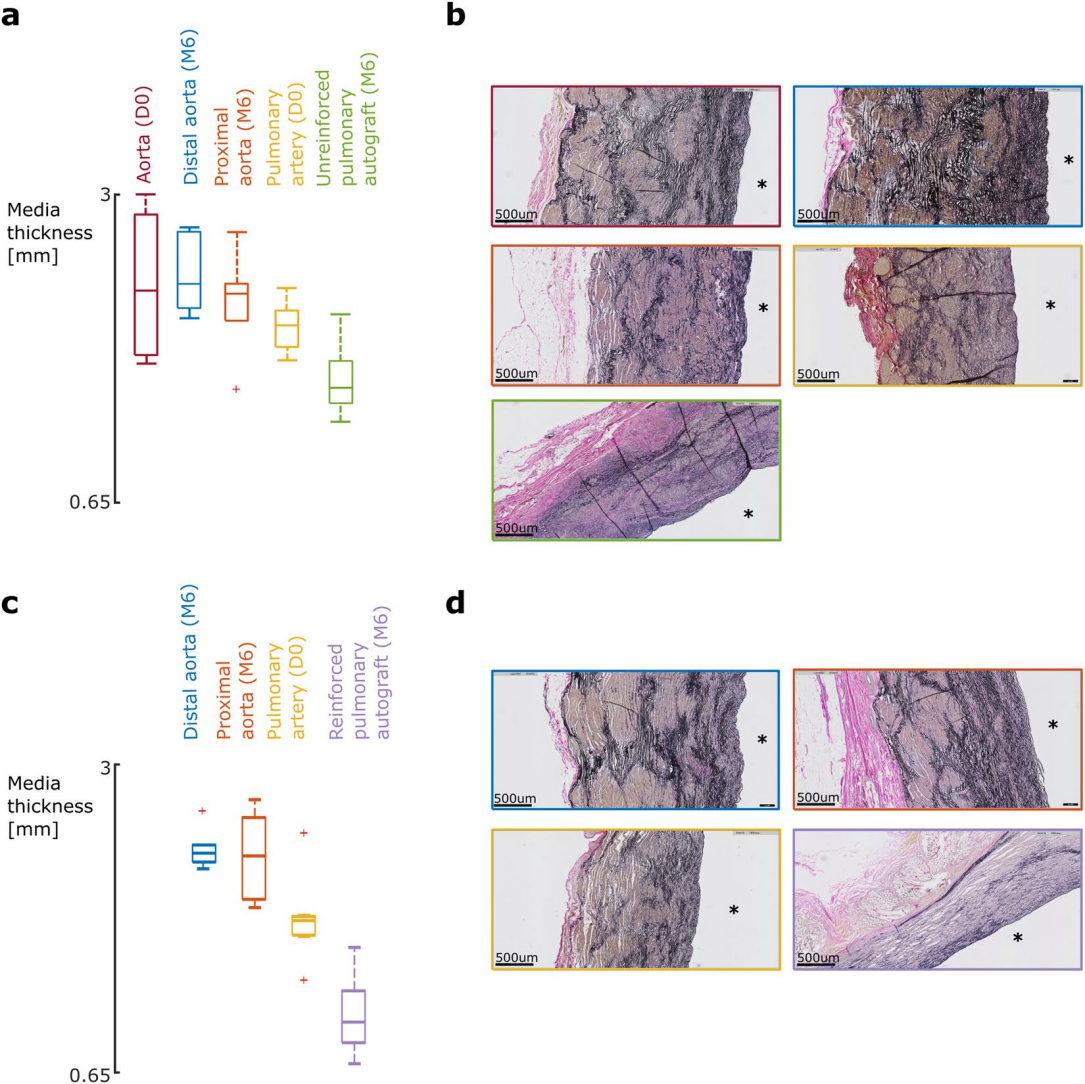
Media thinning as a result of SMC atrophy. **(a,c)** Media thickness data of the UPA group **(a)** and RPA group **(c)**. Histological examination of the autograft reveals significant thinning of the tunica media in both the UPA and RPA group due to atrophy of the SMC as depicted in **(b,d)** (p = 0.041, p = 0.00011). The media thinning is more prominent in the reinforced samples. **(b,d)** Transverse microscopic sections of native aorta (red), distal aorta (blue), proximal aorta (orange), native pulmonary artery (yellow), unreinforced pulmonary autograft (green) and reinforced pulmonary autograft (purple). Elastica Van Gieson stain, original magnification x5. The luminal side is marked with an asterisk.

The mean native aortic media thickness of the UPA group is 2.3 ± 0.6 mm. Six months later, similar media thicknesses are measured proximal (2.1 ± 0.4 mm) and distal (2.4 ± 0.3 mm) to the pulmonary autograft. The mean native pulmonary artery media thickness is 2.0 ± 0.2 mm. After exposing this vessel to aortic pressures for six months without reinforcement, the media thickness decreases significantly (1.6 ± 0.3 mm, p = 0.041) (Fig. 7a,b). The decrease in pulmonary autograft media thickness is more prominent in the reinforced group (1.8 ± 0.3 mm versus 1.1 ± 0.3 mm, p = 0.00011) (Figs. 7c,d).

**Figure 8:**
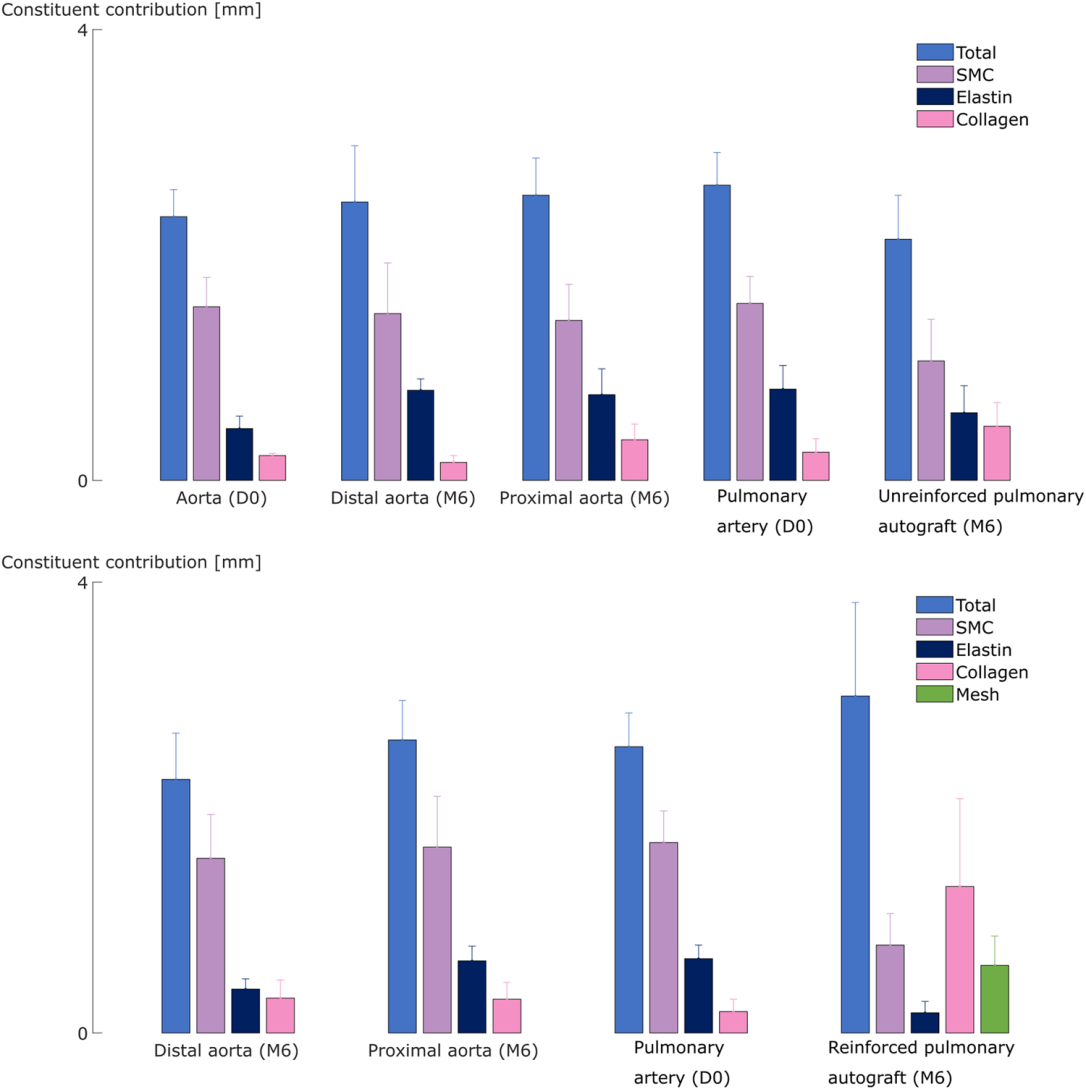
Biomechanical-induced and inflammation-induced collagen deposition. The graphs represent the constituent contribution to the thickness of the tissues of the UPA group (top) and RPA group (bottom). Atrophy of the SMC is seen in all of the pulmonary autograft samples of both the UPA and RPA group, however more prominently in the latter. In the UPA sheep, the amount of collagen fibers of the pulmonary artery increases twofold after reimplantation. In the reinforced pulmonary autograft samples, the mesh is well incorporated with a collagen sheet.

Figure 8 (top) represents the vascular wall microstructure in the UPA samples. Atrophy of the SMC is present in all UPA samples (p = 0.048). The elastin fibers appear intact, yet their characteristic undulations are lost in some regions. The amount of collagen fibers increases twofold, from 0.2 mm to 0.5 ± 0.2 mm, with an increase range of −0.1 mm to +0.4 mm (Figs. 3c and 4d). The wall microstructure of the native aorta and the aorta proximal and distal to the autograft remain unchanged.

Adding a macroporous reinforcement results in a more prominent loss of SMC in the pulmonary autograft (p = 0.00023) (Fig. 8 (bottom)). The elastin fibers appear thinner and stretched out, and to a lesser extent, fragmented. Accordingly, the elastin quantity is reduced from 0.4 ± 0.1 mm to 0.2 ± 0.1 mm (p = 0.00049). The mesh is incorporated in the vessel wall with a fibrotic sheet consisting of collagen fibers, fibroblasts, neovessels and foreign body giant cells. In case of a non-perfect fit of the mesh during surgery, the gap is filled with oedema (Fig. 4d).

## Discussion

It is well-known that arterial tissues adapt when exposed to a new mechanical environment and many efforts are undertaken to model the processes that take place during this adaptation^14^. However, experimental data evaluating what happens both on a mechanical and histological level is lacking^22^. By creating two different mechanical environments, *i.e*. exposing pulmonary artery to systemic conditions with and without reinforcement, we aimed to fill this experimental gap.

### Effect of systemic conditions on autograft dilatation

The diameter increase directly after implantation of the pulmonary autograft is clearly shown in Fig. 2a. This dilatation is due to the pressure increase which is up to sevenfold greater in the aorta in physiological conditions^23^. The UPA continued to increase in diameter at 2 and 6 months, which is consistent with the autograft dilatation occurring in patients who underwent the Ross procedure^3^. Ando *et al*. concluded that autograft dilatation in Ross patients is accompanied by a progressive increase in stiffness. We could not confirm this observation based on Fig. 2b, where the change in diameter throughout the cardiac cycle remains similar for the UPA for the three timepoints.

Reinforcing the pulmonary autograft allows a further increase in diameter of the autograft until two months. In accordance with Izgi *et al*., adding this type of macroporous mesh around the dilated aortic root of Marfan patients prevents further root dilatation^24^. It also decreases the change in diameter occurring throughout the cardiac cycle.

The diameter change for the proximal and distal aorta is different in the UPA group, the proximal change being higher. This corresponds to findings of Kruger *et al*. who found that compliance decreases along the length of the aorta^25^. In the RPA group, this difference is no longer present, related to the mesh extending beyond the pulmonary autograft in some sheep and most likely hindering movement of the surrounding tissue.

### Effect of systemic conditions on the mechanical properties

When categorizing the UPA samples based on the strain they were able to reach in the biaxial tensile test (Fig. 5), we observe two distinct groups: samples that stiffen at low strain (<1.2), showing more aorta-like behavior, and samples that stiffen later (>1.2), behaving like native pulmonary artery (Figs. 3 and 5). The former samples remodeled due to the altered mechanical conditions, whereas the latter did not adapt. Figure 3 demonstrates that in some autografts, both scenarios occur. This might be explained by the asymmetry in the shape of the pulmonary autograft *in vivo* (Fig. 1). Most likely, the two samples came from locations where the *in vivo* loading was distinctly different, either triggering or failing to trigger remodeling processes. This conclusion cannot be drawn for all sheep; sometimes all samples were able to remodel, whereas in other sheep, all samples retained stress-stretch curves similar to pulmonary artery. We propose two reasons for this discrepancy. First, not all pulmonary autograft samples are taken from the same location for each sheep. Second, when placing the pulmonary autograft in aortic position, the axial prestretch imposed was different per sheep.

Looking at Figs. 4 and 6, the same categorization can be made for the RPA samples. Since the tissue-mesh-complex was tested, we would expect the displayed stress-stretch curves to be dominated by the mechanical behavior of the macroporous mesh. We did not test the mesh separately as our testing method cannot handle porous meshes. Looking at the slopes up to 20%, the RPA slopes are indeed considerably higher than those of native pulmonary artery, most probably due to the behavior of the mesh. In the samples that were stretched further, the slopes in the higher strain regions resemble the slopes of native pulmonary artery. This could be related to detachment of the mesh at higher stretches in areas of poor incorporation, such that only the native tissue is tested from that point forward.

The average maximal 1^*st*^ Piola-Kirchhoff stress a sample experienced before failure serves as a proxy for tissue strength. In the autograft samples, only the longitudinal direction was tested which included the anastomoses. Failure first occurred in the anastomoses rather than within the autograft tissue. The UPA strength is slightly lower than that of native pulmonary artery. Since failure always occurred at the anastomosis, this reduction is due to the surgery rather than the remodeling. Adding a reinforcement increases sample strength.

### Effect of systemic conditions on the microstructure

From a histological point of view, exposing the pulmonary artery to systemic conditions led to three major changes. First, a decrease in media thickness due to atrophy of the SMC was measured in every UPA sample (Fig. 7). This is in contrast with the results of Schoof *et al*., who implanted the pulmonary artery in the ascending aorta of pigs for 10 months and reported an increase in large SMC, frequently oriented more longitudinally^1^. Second, the elastin fibers appeared more stretched as a direct consequence of the dilatation. Schoof *et al*. also reported medial elastin fragmentation in failed pulmonary autografts after the Ross procedure^2^. The final histological change was an overall doubling of the amount of collagen fibers (Fig. 8), which is consistent with several studies of similar situations: aortic remodeling following hypertension is characterized by an exuberant, inflammation-driven deposition of collagen in the adventitia^18–21^, and failed pulmonary autografts after the Ross procedure are characterized by increased collagen content^2,8,15^. The latter studies also reported variable intimal thickening, as was reported in pulmonary blood vessels of pulmonary hypertensive patients^16,17^. This was, however, not present in our pulmonary autograft samples. Chiarini *et al*. compared the proteome of pulmonary autograft samples of nine patients reoperated after the Ross procedure with pulmonary artery and aorta samples of seven cardiac donors. The proteomes were distinctly different, featuring *e.g*. the upregulation of proteins related to focal adhesions and metalloprotease-regulating proteoglycans and the downregulation of proteins controlling elastic fiber^26^.

The RPA was characterized by more pronounced SMC loss and stretched elastin fibers (Fig. 7). These results are in line with our previous studies^27–30^. In contrast, a patient study examining the capacity of the macroporous material to withstand further dilatation of a weakened vessel wall does not mention atrophy of the SMC^31^. However, the latter was a study of the aortic root of a single Marfan patient, which has a low number of SMC to start with.

Overall the meshes seemed well incorporated in all sheep, again in line with our previous studies^27–30^. Nevertheless, since the mesh material was not personalized for each surgery, some areas presented gaps between the mesh and the native wall which were filled with edema or proliferating, but inactive SMC. This is in agreement with the findings of the mechanical tests, where certain samples seemed to have limited mesh incorporation (Fig. 4).

### Bringing it all together

Placing the pulmonary autograft in systemic conditions increases the diameter of the autograft. Together with an increase in axial prestretch, this causes the pulmonary autograft to operate in the higher strain region on its stress-strain curve. Consequently, the constituents within the arterial wall experience a mechanical environment that deviates from homeostasis.

Our data shows that these non-homeostatic conditions always trigger SMC loss and stretched elastin fibers. In certain locations of several UPA, this also triggers collagen fiber deposition, which seems to translate in a more aorta-like mechanical behavior (green line in Fig. 3). This is consistent with Bakris *et al*., who showed that an increase of collagen deposition directly stiffens the vascular wall^32^.

On the other hand, we also saw UPA samples that showed native pulmonary behavior, which probably correspond to the histology samples without increased collagen deposition. In fact, in one sheep, the autograft even showed collagen decrease and none of its mechanically tested pulmonary autograft samples showed remodeled behavior. Our surgical data revealed that this specific autograft was implanted with an axial prestretch almost three times as high as the native axial prestretch, compared to the mean axial prestretch of 1.3 of the other autografts. We hypothesize that extreme mechanical overloading damages the mechanosensing pathways, disabling a mechanics-driven increase in collagen production and in severe cases even resulting in enhanced degradation of extracellular matrix.

### Limitations and future work

This study brings unprecedented quantitative insight into remodeling phenomena in large arteries in an *in vivo* context. Nevertheless, *in vivo* longitudinal experiments always pose logistic challenges which have led to some important limitations. First, the pulmonary artery was placed as an interposition graft in the descending aorta. However, in the clinical examples cited in the introduction, the pulmonary artery is reimplanted in the ascending aorta. The design of the experimental animal model posed a challenging trade-off between creating a clinical realistic model and creating a reproducible model of exposing pulmonary arterial tissue to systemic conditions. Therefore, we performed the surgery in female, adult sheep and avoided the close presence of the aortic valve. We consider the minor differences in flow and blood pressure between the ascending and descending aorta to be negligible compared to the differences between pulmonary artery and aorta.

Second, we see a large spread on our results, especially noticeable in the two groups observed in the stress-stretch curves, indicating that the mechano-biological remodeling of the pulmonary autograft shows a large inter- and intra-specimen variability. This variability can be attributed to slight variations in the surgical procedure, especially in terms of axial prestretch as well as in terms of precise autograft shape. Due to the shape of the pulmonary artery, the autograft was wedge-shaped rather than cylindrical, and the angle of the wedge showed variations per sheep. Moreover, one side of the autograft was supported by the spine, exacerbating the inhomogeneity in mechanical loading situation. Unfortunately, we don’t have extra information on the exact location from which each of the samples were taken, making it difficult to prove the correlation between local loading situation and remodeling behaviour. In addition, we were not able to link the mechanically tested samples to their exact histological equivalent.

To resolve these limitations, we see multiple directions for future work. First, numerical simulation of the interposition, using the exact geometries and correlating with the MRI images, will allow to obtain insight into the loading distribution over the autograft. Furthermore, using the flow data collected at the follow-up MRIs, computational fluid dynamics simulations can also bring insight into the effect of altered wall shear stress. Augmenting these simulations with growth and remodeling models as in^14,33,34^ enable to predict how a mismatch between homeostatic loading and current loading triggers remodeling. The dataset collected in the current study can then be used to verify assumptions made within these *in silico* simulations. Second, analogous experiments in a more controlled environment, *e.g*. in a bioreactor, can be performed with a focus on obtaining a homogeneous loading pattern.

## Conclusion

Exposing the pulmonary artery to a systemic environment without adding a reinforcement leads to a progressive dilatation. This is also reflected in the mechanical and microstructural characteristics. Several pulmonary autograft samples displayed aorta-like mechanical behavior, whereas some samples were not able to adapt to the new environment and remained mechanically similar to native pulmonary artery. On a microstructural level, it was clear that the pulmonary artery adapted to the new environment by increasing collagen deposition. However, atrophy of SMC and a decrease in media thickness also occurred.

Adding a macroporous reinforcement to the autograft halted progressive dilatation, but decreased *in vivo* compliance. Loss of SMC was more pronounced and the mesh was well incorporated. This incorporation was reflected mechanically by initially higher slopes in the stress-strain curves.

In conclusion, this study documents remodeling processes of arterial tissue in a relevant *in vivo* setting, quantifying the observed phenomena visually, mechanically and histologically. This valuable dataset can be used to advance fundamental understanding and improve *in silico* modeling of arterial growth and remodeling.

## Materials and Methods

### Surgical procedure

The animal experiments were approved by the Animal Ethics Committee of KU Leuven (P135/2016) and performed according to the regulations of the competent animal welfare agencies. A pulmonary artery interposition graft was placed in aortic position in 17 female Swifter sheep as described previously^27^. Sheep were sedated with an intra-muscular injection of ketamine (15 mg/kg). Anesthesia was induced and maintained with isoflurane (5% and 2–3%, respectively). The pulmonary artery was carefully exposed through a left thoracotomy. With a right atrial to pulmonary artery bifurcation bypass, about 1 cm of pulmonary artery (the native pulmonary valve excluded) was resected and replaced with a prosthetic graft (diameter 18 mm). The resected pulmonary artery was relocated as an interposition graft in the descending aorta. The interposition graft dilated immediately after removing the passive shunt between the ascending and descending aorta, due to exposure to higher pressures compared to the native situation. In eight sheep, the pulmonary autograft was left unreinforced (UPA group), whereas in nine sheep, a macroporous polyethylene terephthalate mesh (Exstent Ltd., Tewkesbury, UK) was placed around the autograft after applying systemic pressure (RPA group). The native pulmonary arterial prestretch was registered during surgery. The axial prestretch imposed on the pulmonary autograft was tracked by measuring the distance between two markers before explantation, after explantation and after reimplantation.

### ECG-gated MRI

Follow-up consisted of three ECG-gated MRIs, at day four, week eight and month six (Fig. 2a). Each time, apart from a 3D angiography, 2D MRI's over the cardiac cycle were taken at 3 specific locations to measure flow and diameter through time. The selected transversal planes were located proximal and distal to the autograft and at the center of the autograft (as indicated in Fig. 2b). At each location, 20 diameters throughout the cardiac cycle were obtained by applying a simple thresholding procedure to extract the lumen and deriving the diameter from a circle with equivalent surface area.

### Mechanical analysis

Samples were stored in phosphate-buffered saline (PBS) at −80 °C prior to analysis. Two types of mechanical experiments were conducted: planar biaxial experiments and uniaxial tensile tests. The following tissue types were harvested: native pulmonary artery, aorta proximally and distally located with respect to the autograft, and pulmonary autograft. Tissues were divided into samples and prepared for either planar biaxial testing or uniaxial tensile testing.

### Planar biaxial experiments

Planar biaxial testing was performed to obtain stiffness-related properties. Squared samples of 10 mm by 10 mm were excised from the obtained tissues. The thickness was measured optically with a Canon EOS450D. A speckle pattern was created by gently laying the sample on a paper with scattered graphite powder. Next, the sample was mounted in a MessPhysik biaxial tester using four sets of five rakes with a thickness of 0.3 mm and a spacing of 0.7 mm. During testing, the sample was submersed in PBS at 37C.

The test protocol consisted of four loading steps (with a nominal strain level based on the distance between the rakes of $\mathrm{5 \% }$, $\mathrm{25 \% }$, $\mathrm{50 \% }$ and $\mathrm{75 \% }$) at $\mathrm{5 \% /}s$. Each loading step contained five ratios between the circumferential and longitudinal direction: $\mathrm{1:1}$, $\mathrm{1:0.5}$, $\mathrm{1:0.25}$, $\mathrm{0.5:1}$ and $\mathrm{0.25:1}$. Each stretch cycle was repeated five times for preconditioning and a preload of 50 mN was imposed before each stretch cycle. The stretch-phase of the last cycle of the 1:1 ratio of the final loading step was selected for analysis.

The deformation was tracked using digital image correlation (DIC, Vic2D, Correlated Solutions, Irmo, USA). The area of interest for DIC was selected as the central $\mathrm{25 \% }$ area between the rakes, where stress and strain are assumed to be more uniform^35^. The subset size was taken as 29 with a step size of 7. Based on the measured deformation gradient using DIC, the stretch in both directions of the test was calculated.

The forces of the two pairs of opposing load cells were averaged and used to calculate the first Piola-Kirchhoff stress, by dividing the averaged force in each direction ($R{F}_{ii}$) by the initial loaded area ${A}_{\mathrm{0,}i}$. The latter was calculated by multiplying the thickness with the average distance between the rakes.

Each of the performed mechanical tests results in a stress-stretch curve. In order to concisely show all results, each of these curves was approximated by its slope at the $\mathrm{5 \% }$, $\mathrm{10 \% }$, $\mathrm{15 \% }$, $\mathrm{20 \% }$, $\mathrm{25 \% }$, $\mathrm{30 \% }$, $\mathrm{35 \% }$ and $\mathrm{40 \% }$ stretch level. This was calculated by fitting a straight line through the points between $i-\mathrm{2.5 \% }$ and $i+\mathrm{2.5 \% }$ for each stretch level $i$, which results in a local stiffness measure at each of these levels. These slopes were then averaged over the different stress-stretch curves for each tissue type.

### Uniaxial tensile tests

Uniaxial tensile testing was performed to obtain failure properties. For the aorta and native pulmonary artery, dogbone-shaped samples oriented in circumferential and longitudinal direction were prepared with a neck width of 3 mm. For the autograft samples, both reinforced and unreinforced, rectangular samples with a width of 5 mm were prepared. The latter samples were always collected in longitudinal direction around the anastomosis, *i.e*. the transition between the autograft and the aorta, such that the center of the sample contained this anastomosis. After mounting the sample in mechanical clamps, the sample was cyclically loaded five times up to $\mathrm{25 \% }$, $\mathrm{50 \% }$ and $\mathrm{75 \% }$ consecutively, at $\mathrm{5 \% /}s$, and with a preload of $0.03$ N before each cycle. After these 15 cycles, the sample was stretched until failure. The latter was used for further analysis.

The forces of the two opposing load cells were averaged and used to calculate the first Piola-Kirchhoff stress, by dividing the averaged force ($R{F}_{uni}$) by the initial loaded area ${A}_{uni}$. The latter was calculated by multiplying the thickness with the initial width in the neck region.

### Histological analysis

Transversal samples of aorta, reinforced and unreinforced pulmonary autograft and native pulmonary artery were fixated in $\mathrm{6 \% }$ paraformaldehyde, dehydrated and embedded in paraffin in *ex vivo* configuration. 6-*μ*m-thick serial cross-sections were prepared (Microm HM360) and stained through Hematoxylin and Eosin, Picrosirius Red and Elastica van Gieson stains. A Nikon Eclipse C1 microscope with NIS elements (Version 4.60) imaging software was used to examine the samples. Total wall thickness and layer-specific thicknesses were measured five times per sample with ImageJ. The percentages of elastin, collagen, SMC, bleeding, fibrotic sheet, and mesh were determined three times per sample using in-house developed image processing software in Matlab r2018b using K-means clustering and detection of pixels with similar RGB-values. These percentages were multiplied by the total wall thickness to facilitate comparison between different tissues and is further referred to as constituent contribution (which is consequently expressed in mm since fractions are dimensionless and wall thickness has mm as unit).

### Statistics

Data were analyzed using Matlab R2018b (MathWorks Inc., Natick, MA, USA) and Microsoft Office Excel (Microsoft Corp., Redmond, WA, USA). Results are expressed as mean ± standard deviation. A p-value < 0.05 was considered statistically significant. Variables were compared using the repeated measures ANOVA test (when comparing longitudinal data over time, e.g. MRI data) and the two sample t-test (when comparing two groups at one point, e.g. mechanical and histological data).
